# Real-Time Streaming of Surgery Performance and Intraoperative Imaging Data in the Hybrid Operating Room: Development and Usability Study

**DOI:** 10.2196/18094

**Published:** 2020-04-22

**Authors:** Chun-Cheng Lin, Yu-Pin Chen, Chao-Ching Chiang, Ming-Chau Chang, Oscar Kuang-Sheng Lee

**Affiliations:** 1 Institute of Clinical Medicine National Yang-Ming University Taipei Taiwan; 2 Division of Orthopaedic Trauma Department of Orthopaedics and Traumatology Taipei Veterans General Hospital Taipei Taiwan; 3 Department of Orthopedic Surgery Wan Fang Hospital, Taipei Medical University Taipei Taiwan; 4 Department of Surgery National Yang-Ming University Taipei Taiwan; 5 Department of Orthopaedics and Traumatology Taipei Veterans General Hospital Taipei Taiwan; 6 Department of Medical Research Taipei Veterans General Hospital Taipei Taiwan

**Keywords:** hybrid operating room, real-time streaming, surgical telementoring, information technology infrastructure, encoder and decoder, real-world evidence, information technology, surgery, medical imaging, operating room

## Abstract

**Background:**

The trend of quick evolution and increased digital data in today’s operating rooms (ORs) has led to the construction of hybrid ORs. There is often a main control room with monitors for integrating intraoperative data from multiple devices in the hybrid OR. However, there is no adequate solution for communicating the data with people outside the OR.

**Objective:**

The objective of this study was to design an intelligent operating room (iOR) system, augmented onto the existing information technology (IT) infrastructure of hybrid ORs, to stream surgery performance and intraoperative imaging data.

**Methods:**

In this study, an all-in-one device with synergetic encoder and decoder was used. The device was able to stream multiple sources to one display. The lossless video and images from specific surgical workflows were streamed outside the hybrid OR through network protocols and were further managed by a streaming server and wireless control system. The steps of this study included the following: (1) defining the requirements and feasibility of an iOR system in the hybrid OR, (2) connecting multiple sources, (3) setting up equipment across the hybrid OR and a conference room, (4) designing a video management system, and (5) real-time streaming under specific surgical workflows.

**Results:**

The wired streamed video was shown simultaneously on the display in the hybrid OR and the display in the conference room with near-zero latency. Additionally, an interactive video between the hybrid OR and the conference room was achieved through the bidirectional wireless control system. The functions of recording, archiving, and playback were successfully provided by the streaming server. The readily available hardware components and open-access programming reduced the cost required to construct this streaming system.

**Conclusions:**

This flexible and cost-effective iOR system not only provided educational benefits, but also contributed to surgical telementoring.

## Introduction

### Background

Health information management is crucial for hospitals. In surgical departments, today’s operating rooms are evolving quickly, and large amounts of digital data, including images and videos, are produced every day. The hybrid operating room (OR) is defined as being equipped with advanced medical devices such as fixed C-arm fluoroscopy, cameras, a computed tomography (CT) scanner, or a magnetic resonance imaging (MRI) scanner [1]. In addition to these imaging and video devices, there is often a main control room with monitors for integrating intraoperative data. However, there is currently no adequate solution for communicating the data from the hybrid OR to people outside the OR.

Currently, manufacturers build integrated OR infrastructures that provide video acquisition, storage and routing of continuous video data within the OR, although most of them are not based on existing OR information technology (IT) infrastructure [2]. Other drawbacks to OR setups of this kind include a lack of streaming outside the OR, the need for high-cost customized software for video management, and difficulty in integration with new devices of different brands, such as an incompatibility between imaging and surgical navigation systems.

With regard to streaming surgery performance outside the OR, previously published studies discussed a system that could integrate data from ceiling cameras and a vital sign monitor [3]. However, in the hybrid OR, receiving data from multiple sources results in more difficulties for real-time integration and there is unmet need in this regard.

### Objectives

We aimed to establish a framework based on the IT infrastructure of the hybrid OR, and to stream data through Ethernet methods between the hybrid OR and the conference room. The augmented system was named the intelligent OR (iOR) system. There were two streaming object categories: surgery performance and intraoperative imaging data. Furthermore, we aimed to establish a video management system (VMS) for the iOR system, with two kinds of control methods: (1) a wired server named the iOR box and (2) a bidirectional tablet controlled through a wireless connection.

### Hypothesis

The workable, highly flexible, and bidirectional iOR system enables collaboration across the hybrid OR and conference room by making it easier to stream high-definition and near-zero latency data of surgery performance and relative imaging data. The system may significantly lower the costs involved compared to dedicated streaming systems by commercial brands.

## Methods

The iOR system was installed in the hybrid OR in the Yuanlin Christian Hospital, Yuanlin City, Changhua County, Taiwan. The installation of research equipment was approved by the hospital’s administration prior to the commencement of the project.

### Defining the Requirements and Feasibility of the iOR System in the Hybrid OR

To understand the surgical workflow, we carried out a survey of the equipment in the hybrid OR, including existing hardware and software, the demand for infrastructure for streaming open surgeries and endoscopy surgeries, and communication between the vendors and surgeons. The main system in this hybrid OR received image or video data from various sources, including videos from cameras and the endoscopy system, digital data from vital sign monitors, imaging data from portable fluoroscopy, and the picture archiving and communication system (PACS) connected to the Department of Radiology. These devices contributed to two categories of streaming data: real-time surgery performance and intraoperative imaging data. A suitable way of connecting an equipment interface and transporting line between the hybrid OR devices and the iOR system should be arranged. The ideal control systems would have both wired and wireless control systems. All materials in this study, including hardware and software, should be compared with existing products available from commercial brands.

### Connecting Multiple Sources

When researching how to integrate multiple data sources in the hybrid OR, the key to achieving streaming effectiveness was to use an all-in-one device with synergetic encoder and decoder. This device was named NDcoder to indicate it is an encoder and decoder in one single product. We proposed the resolution of streaming all data out of the hybrid OR by using the principle of “one NDcoder connecting one data source”. These devices are available commercially. Multiple NDcoders (SigmaXG, Technolution BV) were used to connect different sources from multiple devices in the hybrid OR and simultaneously transport data over an Ethernet connection. All devices in the hybrid OR could be directly or indirectly connected to an NDcoder through a digital video interface (DVI). The adapter cable was selective.

### Setting Up Equipment Across the Hybrid OR and the Conference Room

**Figure 1:**
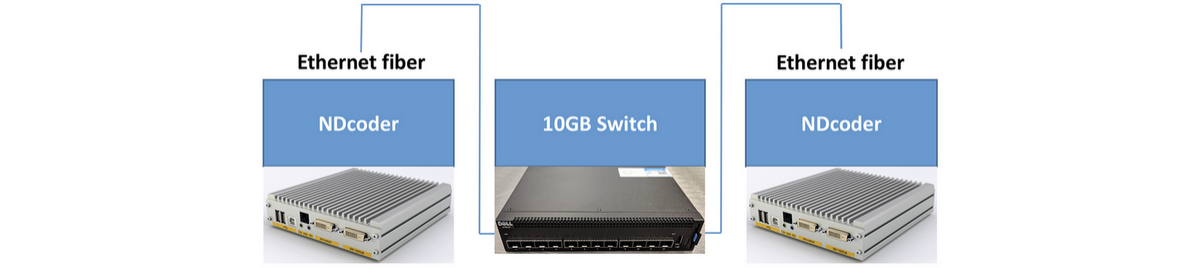
The basic structure of the intelligent operating room system.

One SigmaXG in the hybrid OR shared streaming data to an external NDcoder by indirectly bridging these two devices through Ethernet fibers connected with a 10 GB switch (Dell, Inc). These devices together formed the basic structure of the iOR system that bridged the hybrid OR and a conference room (Figure 1).

### Designing the Video Management System

**Figure 2:**
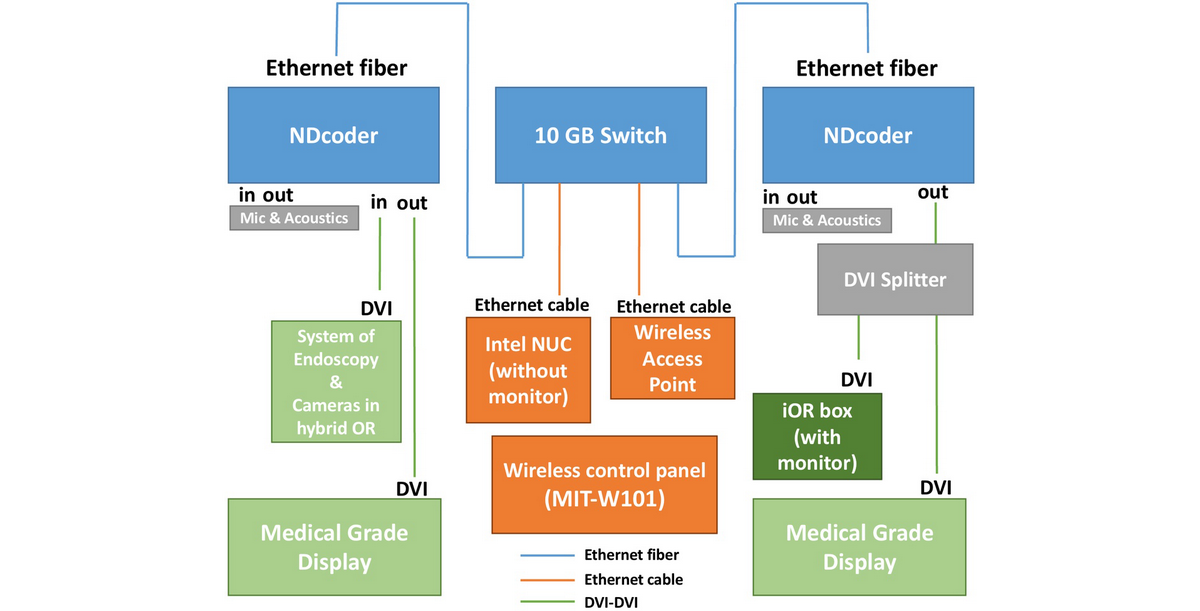
The map of the intelligent operating room system. DVI: digital video interface; iOR: intelligent operating room; NUC: next unit of computing.

The video management system (VMS) included wired and wireless control systems. The main component of the wired control system was the iOR box, which had one video capture card and a connected monitor. Conversely, the wireless control system included three components (shown in Figure 2): a streaming console (Intel Next Unit of Computing [NUC], Intel Inc), a wireless access point (WAP; ASUS Inc), and a wireless control panel (MIT-W101; Advantech Inc).

One DVI splitter was connected to the SigmaXG in the conference room through a DVI cable, enabling data to be transported via DVI cables from the SigmaXG to the iOR box (with monitor). The iOR box with monitor represented the streaming server. The Intel NUC (without monitor) and the WAP in the wireless control system were directly connected to the 10 GB switch through Ethernet cables. Additionally, the wireless panel controlled the Intel NUC through the WAP. This wireless control system served as a communicator between the two SigmaXGs in the iOR system (Figure 2).

One medical grade display was connected to another output interface through the DVI cable. In addition to the data streamed directly from the hybrid OR, the display was able to receive videos or images controlled by the MIT-W101. To allow bidirectional communication, the iOR system had recording (microphones) and playback devices in both the hybrid OR and the conference room (Figure 2).

### Real-Time Streaming Under Specific Surgical Workflows

**Figure 3:**
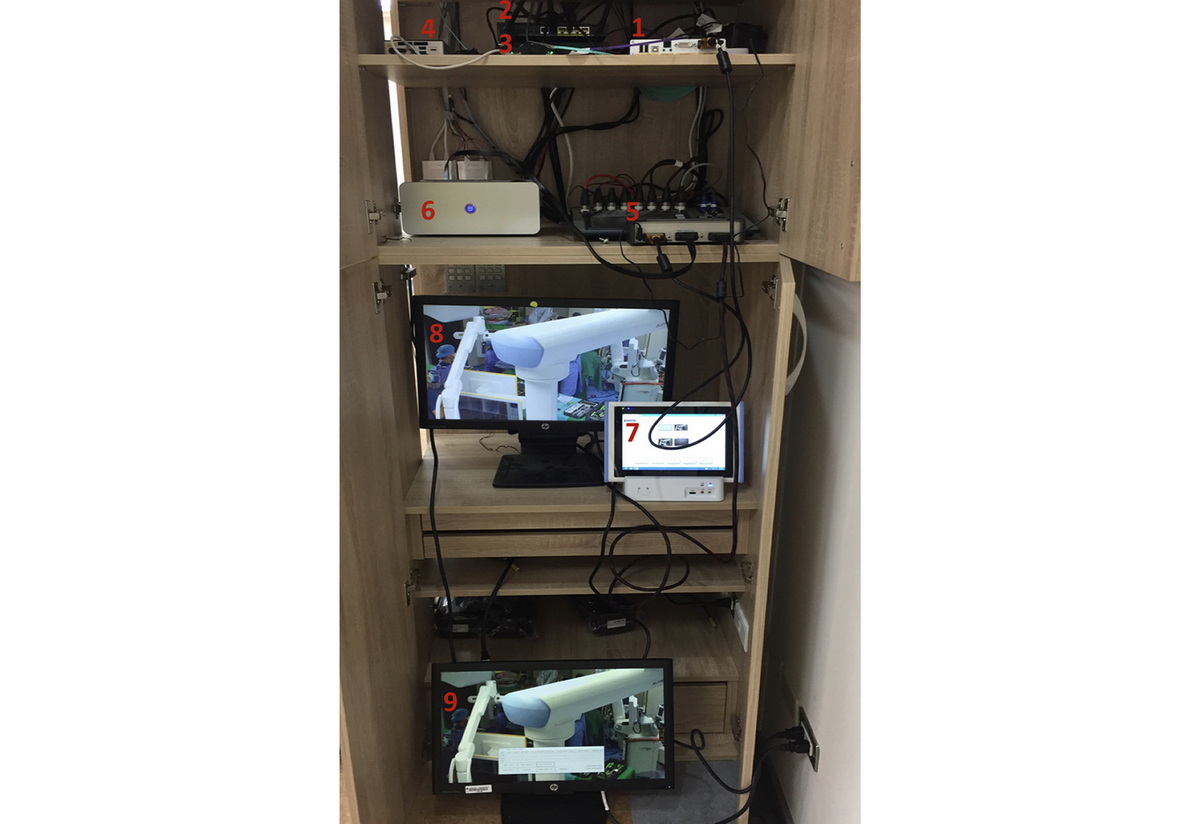
The equipment required for intelligent operating room streaming. (1) NDcoder (SigmaXG), (2) wireless access point, (3) 10 GB switch, (4) Intel NUC, (5) DVI splitter, (6) intelligent operating room box, (7) wireless control panel (MIT-W101), (8) medical grade display, (9) monitor for intelligent operating room box. The NDcoder was connected with the equipment in the conference room by one DVI splitter, while two other NDcoders were installed in the operating room (not shown). DVI: digital video interface; NDcoder: encoder and decoder.

In the hybrid OR, in addition to the devices connected to the NDcoders, there was a display connected to the NDcoder’s output interface with a DVI cable. Thus, the medical staff in the OR were able to see the streaming results on the display. One temporary shelf (Figure 3) was built for the iOR system, and the streamed videos were played in the nearby conference room. During surgery, staff observed whether there was any latency in the wired connection (medical grade display in the conference room) or wireless device (MIT-W101 control panel).

For the real-time test, the authors chose a case involving arthroscopically assisted reconstruction of the anterior cruciate ligament. The patient’s privacy was protected, and associated information was delinked according to the ethical review committee guidelines. During the surgery, three steps were defined by the imaging devices of the hybrid OR: (1) preparing, filmed by the fixed camera with 360° angle; (2) surgical approaches, filmed by the camera over the shadowless lamp; and (3) inside the knee joint, filmed by the endoscope of the arthroscopy system. The data streamed from the cameras and the arthroscopy machine were transported via the methods detailed above.

## Results

In this study, data sources included the following: (1) vital sign monitors, (2) ceiling cameras, (3) cameras on the shadowless lamp, (4) system of endoscopy surgery, (5) portable C-arm fluoroscopy, (6) CT scanner, and (7) PACS from the Department of Radiology, showing preoperative image data, and receiving real time uploaded data from the C-arm or CT scanner in the hybrid OR. All of these devices had DVIs, which were able to transport uncompressed imaging data.

When searching for a commercially available streaming system designed for the hybrid OR, we found that the price of one medical grade monitor was higher than the cost of all the equipment used in the iOR system. This could be due to the costs associated with importing the products of an international brand. Furthermore, in the commercial system, the hardware components and the IT infrastructure were supplied by two different companies. The relevant application programming interface (API) was not open access, which also increased the cost of modifying console applications.

The NDcoder, SigmaXG, was able to distribute lossless video without compression. Through the API and software development kit (SDK), it was possible to change the resolution and the frame rate to limit bandwidth, and multiple imaging sources could be shown on one display synchronously. The API for the SigmaXG is open access and documentation can be downloaded from the manufacturer’s website. For the iOR box and the Intel NUC, the SDK programs and relevant console applications were written by software engineers. This device offered seamless switching. Moreover, there was no black frame displayed on the remote display when one input data was streamed outside the hybrid OR. This was because the NDcoder used a frame buffer in the output channels. In direct display mode, the output frame buffer was bypassed, and video input and output were synchronized with a latency of a few video lines.

**Figure 4:**
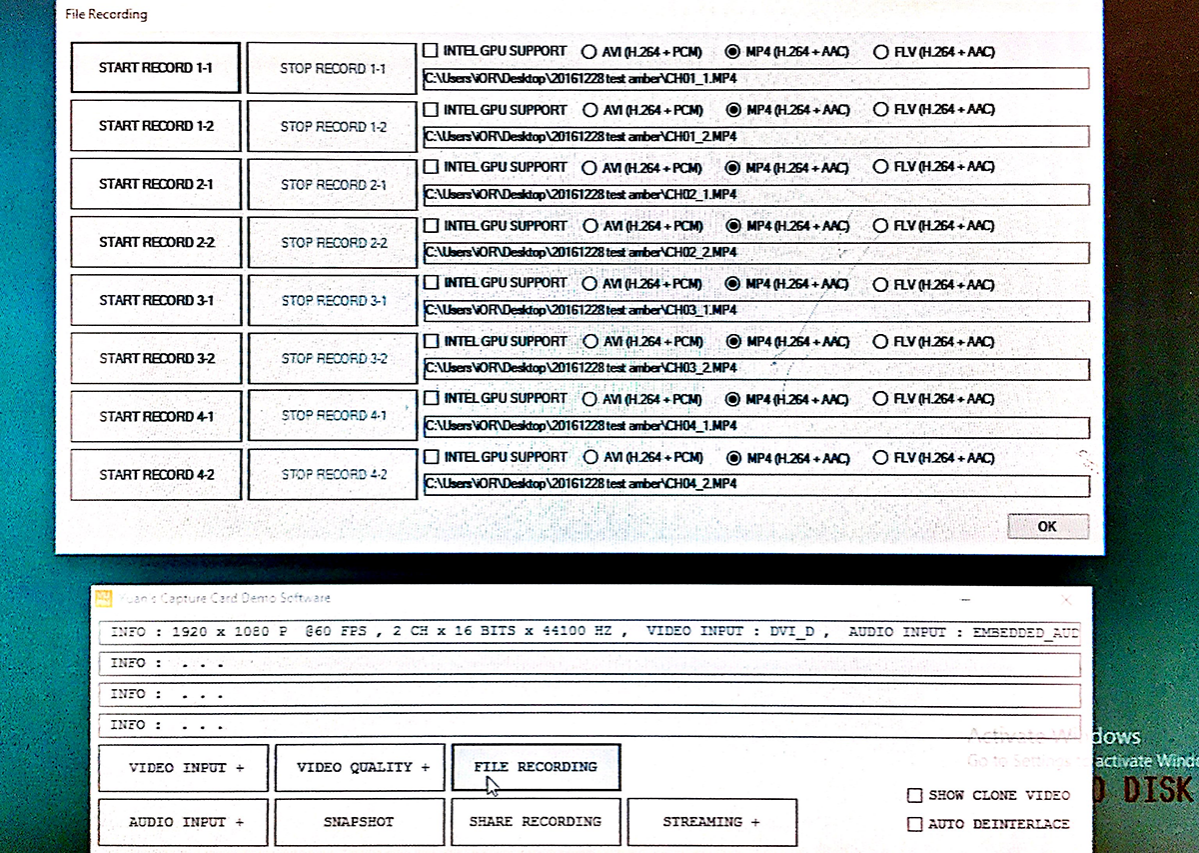
Screenshot of the intelligent operating room box.

The wired streamed video was shown simultaneously on the display in the hybrid OR and on the medical grade display in the conference room. Two different video sources (the camera attached to the shadowless lamp and the endoscope camera) were shown on one display in real time. This streamed data was also captured by the iOR box. The iOR box is able to record, archive, and play back the streamed data (Figure 4).

**Figure 5:**
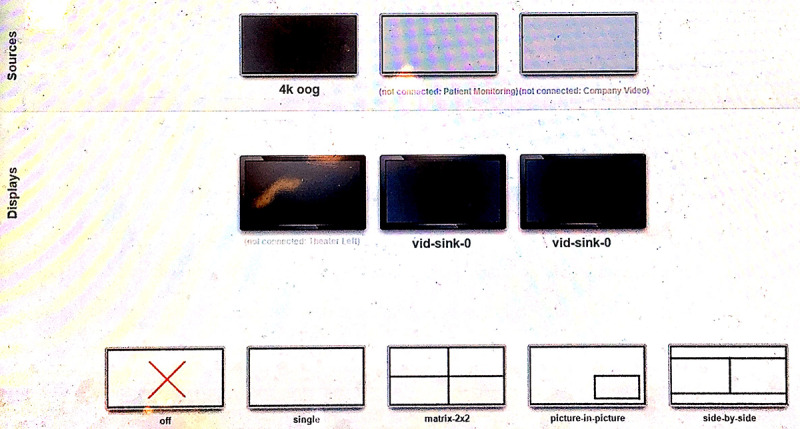
Screenshot of the wireless control panel (MIT-W101).

**Figure 6:**
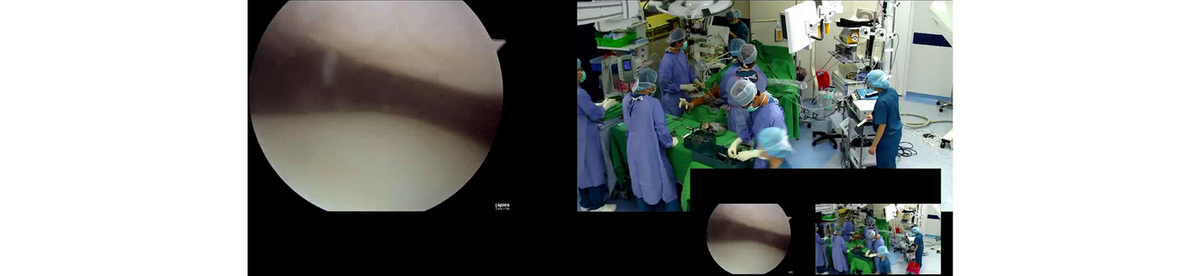
Demonstration of real-time streamed videos of arthroscopy and surgery performance.

The wired streamed video had near-zero latency (60 frames per second with a resolution of 1920×1080 pixels), and the real-time iOR box control system was the data server. Furthermore, an interactive video between the hybrid OR and the conference room could be achieved through the bidirectional wireless control system by using the MIT-W101 control panel and Intel NUC. The wireless streaming console, Intel NUC, was also the server for the wireless system. The software in this wireless control system enabled the MIT-W101 control panel to distribute and split the live or playback screens to the display in the hybrid OR as well as the one in the conference room (Figure 5). However, the wireless video in the MIT-W101 control panel may have some latency. When controlled by the wireless control system, the display in the conference room was able to show the screen in the hybrid OR as a picture-in-picture (Figure 6).

## Discussion

### Overview

Operating rooms are evolving quickly. New medical technologies and minimally invasive procedures have changed the OR environment and how surgeries are performed. Constant changes, increased complexity, and demands for increased productivity mean the surgical suite needs to be more efficient and flexible than ever. Despite lacking data analysis, this study is a proof of concept of the use of a real-time streaming system in a surgical context. The iOR system was intended to have the flexibility of expansion, especially the software functions. This new system includes a bidirectional control system, synergistically wired and wireless systems, and open access API and offers the potential to develop more intelligent applications capable of automatic identification or learning.

### Challenges

Some challenges were encountered in this study. At the beginning, there were many meetings between the technicians and surgeons. Live streaming of multifocal image or video data is typically used for telementoring. Thus, the streamed data needed be collected from different sources and displayed simultaneously in an external location (the conference room). The surgeon was also the director during live streaming. Furthermore, the surgeon had to understand and use both the infrastructure of the hybrid OR and the new iOR system. Additionally, the vendor should have an understanding of how surgeries are performed, as well as the needs of telementoring.

As for streaming itself, streaming surgery performance and intraoperative imaging data is different from streaming for entertainment. Good image quality and a high frame rate are required for some surgeries, such as heart surgery, due to the dynamics of the moving heart. If the streamed data are compressed and encoded, information would be lost in the decoding process. Therefore, the choice of an NDcoder for adequate streaming quality is a very important step when setting up the iOR system, as is the consideration of how to transfer the streamed data.

Internet-based communication solutions rely on high-performance network infrastructure [4]. Gigabyte and terabyte networks are common networks for local and wider area communication. Network protocols such as HTTP, real-time transport protocol (RTP), Zeroconf and File Transfer Protocols have become the foundations of internet communication solutions. In this study, the authors used the 10 GB switch (Dell, Inc) device to connect to the hospital’s network. Thus, the iOR system was an augmentation based on the existing infrastructure, which afforded the network protocol required to stream data.

### Contributions of the iOR System

**Figure 7:**
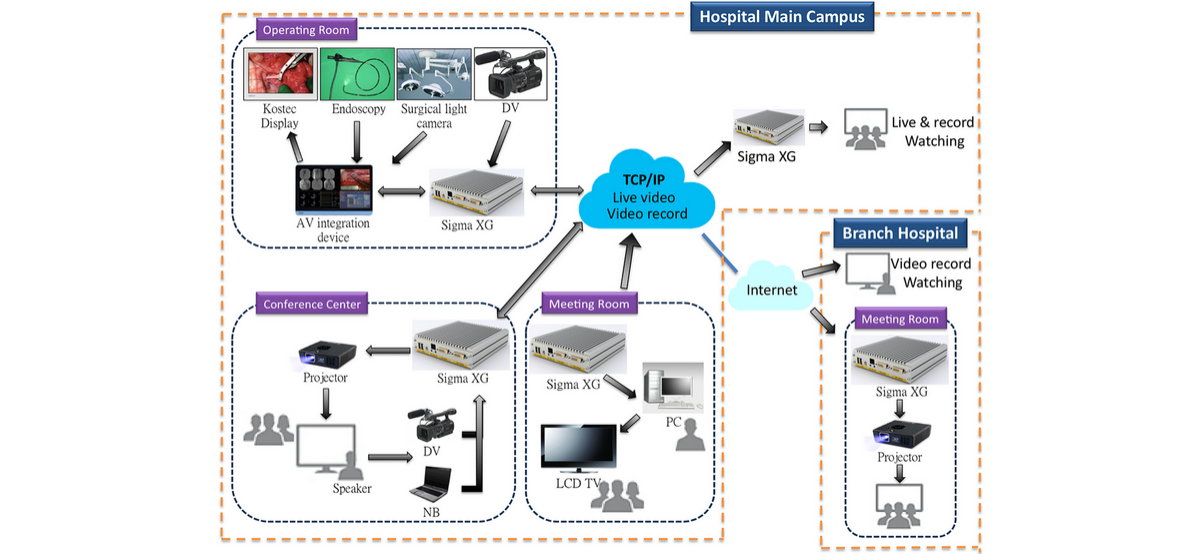
Concept map of the intelligent operating room system. AV: audiovisual; DV: digital video; LCD: liquid-crystal display; NB: notebook; TCP/IP: transmission control protocol/internet protocol.

The iOR system streamed data from the surgical workflow. Since then, the system has promoted the real-time education of training surgeons and has enabled remote supervision. The recorded video in the streaming server (iOR box) could be further designed with an augmented reality (AR) system. Additionally, the wireless system served as the remote control for both live and playback screens. Adding more applications to the iOR system would be flexible and cost-effective (Figure 7).

Streaming data outside the OR is a crucial component of surgical telementoring [5]. Surgical telementoring is one kind of telemedicine, which involves the use of IT to provide real-time guidance and assistance for surgical workflows from a physician at a remote location [6]. In addition to providing an educational advantage, telementoring has the potential to directly provide immediate access to surgical expertise in areas without qualified surgeons. However, the ideal video conferencing methodology should be suitable for streaming. Additionally, telementoring introduces challenges regarding patient security and privacy, and it remains unclear as to how liability would be distributed between the on-site surgeon and mentor. These issues need to be addressed before real-time streaming can become a routinely used tool for surgical telementoring.

### Application of iOR System for Real-World Evidence

The iOR system can support the trend of real-world evidence (RWE), especially in studies involving surgical procedures. The concept of RWE refers to health care data derived from sources outside typical clinical trial settings, including electronic health records [7]. In traditional clinical trials, the population enrolled may be different from those seen by health professionals in daily practice. This is because trials are often conducted with specific populations, in a specialized environment that differs from the clinical reality [8]. In view of this, electronic health records can provide new insight into states of health and illness. To this end, the iOR system is able to gather data and contribute to RWE. Additional issues regarding the feasibility of an iOR system in a traditional OR could present themselves. In the traditional OR, many devices have analog signals which cannot be used to stream data due to an apparent latency. Furthermore, real-time video capture requires additional solutions such as a portable camera station with controlled arms. Audiovisual systems are characterized by extensive cabling and complicated matrix configurations; therefore, digitization and advanced IT construction of traditional ORs is necessary.

### Novel Achievements

The aim of this study was to enable departments with hybrid ORs to stream data outside the OR with easily accessible equipment. Based on the existing IT infrastructure, the cost-effective iOR system described in this study is able to integrate surgery performance with imaging data from high-tech machines.

### Limitations

Although this study was successful as a proof of concept, one limitation is that there was no control group. This study was designed to investigate the feasibility of clinical applications such as live surgeries or live webinars. The results of the study were practical, but there was no comparison to other methods nor quantitative results.

### Conclusions

The real-time iOR system was able to integrate and stream surgery performance and imaging data from existing equipment in the hybrid OR. When using the wired iOR box as the streaming server, the iOR system was able to record, archive, and play back video. Furthermore, the wireless control system manipulated the live or playback screens and further supported collaboration for surgical telementoring, educational conferences, and remote consultations. In the future, a modular prototype will be developed based on the iOR system.

## Abbreviations

API: application programming interface
AR: augmented reality
DVI: digital video interface
iOR: intelligent operating room
IT: information technology
NDcoder: encoder and decoder
NUC: next unit of computing
OR: operating room
RWE: real-world evidence
SDK: software development kit
VMS: video management system
WAP: wireless access point
